# The Development of Small Animal Models for Zika Virus Vaccine Efficacy Testing and Pathological Assessment

**DOI:** 10.4269/ajtmh.16-0277

**Published:** 2016-06-01

**Authors:** Aaron C. Brault, Richard A. Bowen

**Affiliations:** Division of Vector-Borne Diseases, Centers for Disease Control and Prevention, Fort Collins, Colorado; Department of Biomedical Sciences, Colorado State University, Fort Collins, Colorado

Zika virus (ZIKV) is a mosquito-borne flavivirus serologically grouped within the family *Flaviviridae*, which was initially isolated from a febrile sentinel monkey in the Zika Forest of Uganda in 1947. Subsequent genetic analyses have demonstrated the presence of two distinctive African and Asian viral genotypes. After years of being associated only with sporadic human illness, the first outbreak of ZIKV disease was reported in 2007, on the island of Yap in the Federated States of Micronesia (with 49 laboratory confirmed cases documented).^1^ In subsequent years, ZIKV was associated with outbreaks in French Polynesia and then in the South Pacific Islands of New Caledonia and Easter Island, before disease being identified in Brazil in early 2015. Although the majority of human ZIKV infections have been found to result in asymptomatic or mild illness,^1^ the recent identification of an association of ZIKV infection during pregnancy with an increase in the incidence of microcephaly in neonates,^2^ as well as the development of Guillain–Barré syndrome resulted in the World Health Organization's declaration that the ZIKV outbreak constituted a “Public Health Emergency of International Concern.”^3^ The unprecedented geographic expansion of the virus, with up to 1.3 million estimated human infections as of December 2015,^4^ its sexual transmission potential, and its severe pathogenic effects in fetuses (microcephaly, ocular malformations) have emphasized the critical need for available vaccines and antiviral therapies, as well as an improved understanding of the pathological mechanisms that result in human disease after infection with this virus.

In this issue of the *American Journal of Tropical Medicine and Hygiene*, Rossi and others^5^ describe preliminary experiments that have identified murine models of ZIKV pathogenesis. This work explores the pathological response of type I interferon receptor knockout mice (A129) as well as type I and type II interferon receptor knockout mice (AG129) to peripheral challenge with a low-passage Asian strain of ZIKV that was isolated from Cambodia in 2010. A129 mice that were unable to respond to interferon-alpha and -beta signaling manifested high viremia and demonstrated an age-dependent susceptibility to disease. Three week-old mice succumbed to infection and developed high viremia (> 7 log_10_ plaque-forming units/mL sera), whereas older mice exhibited less mortality and lower viremias. Similarly, 3-week-old AG129 mice, unable to respond to type I and II interferon signaling, were inoculated with the same ZIKV strain, and demonstrated mortality after intraperitoneal or intradermal inoculation. Three week-old A129 and AG129 mice succumbed within 7 days of inoculation, with notable neurological involvement and subsequent identification of virus in brain tissue. In contrast, immune-competent mice peripherally challenged with the same ZIKV strain failed to manifest viremia or demonstrate any signs of morbidity.

Most importantly, these immunocompromised mouse models demonstrated disease and viremia after infection with a low-passage ZIKV strain, negating the necessity for passage adaptation for the elicitation of virulence. Previous attempts to develop ZIKV murine models involved the serial passage of the prototype strain in mice,^6^ complicating the assessment of the effects of passage on virulence. Furthermore, passage adaptation can alter the antigenicity of the resultant virus, thus obscuring any assessment of immunization with a circulating strain of ZIKV from which a candidate vaccine is likely to be generated. Although the authors have not directly assessed these models for susceptibility to the specific Asian genotype viruses currently associated with the ongoing outbreak, this work has set the stage for these assessments, and serves as an initial, significant step toward developing tools to assess the safety, immunogenicity, and protective efficacy of ZIKV vaccines under development. Although the use of immunocompromised murine models negates the direct assessment of the role of interferon response elements in the disease process, this communication provides an experimental framework that may be highly useful for the assessment of host and virological factors involved in intrauterine and sexual transmission of ZIKV, in addition to serving as a vaccine challenge model. The authors did not assess specific tissues of the female reproductive tract; however, robust viral replication was observed in the testes of both A129 and AG129 mice, indicating the potential appropriateness of these models for assessing potential mechanisms for sexual transmission.

Data from nonhuman primate studies with ZIKV that have been provided online (https://zika.labkey.com/project/OConnor/ZIKV-001/begin.view) in a real-time manner have demonstrated viral replication of a ZIKV isolate from French Polynesia. However, the development of a small animal model for initial assessment of the safety, immunogenicity, and protective efficacy of candidate vaccines will facilitate the much broader screening of potential vaccine candidates. A more thorough initial stage screening in a rodent model will likely provide more viable candidates for nonhuman primate testing and subsequent human clinical trials. Although the ultimate utility of these two mouse models for recapitulating the pathogenic potential of different ZIKV strains for eliciting disease in humans is currently unknown, this study ushers an important initial advance toward that end, and it probably offers critical initial models for assessing first-generation ZIKV vaccines.
